# Circumferential minimally invasive reconstruction for lumbar destructive deformity secondary to spinal gout: a case report

**DOI:** 10.3389/fsurg.2026.1776837

**Published:** 2026-06-26

**Authors:** Cheng-Chun Peng, Dueng-Yuan Hueng, Wei-Hsiu Liu, Meng-Chi Lin, Bon-Jour Lin

**Affiliations:** 1 Department of Surgery, Zuoying Armed Forces General Hospital, Kaohsiung, Taiwan, Kaohsiung, Taiwan; 2 Department of Neurological Surgery, Tri-Service General Hospital, Taipei, Taiwan

**Keywords:** gout, minimally invasive surgery, oblique lumbar interbody fusion, surgical endoscopy, spinal fusion

## Abstract

Spinal involvement constitutes an uncommon manifestation of gout, and destructive deformity as a consequence of spinal gout is rare. A case is presented involving a 59-year-old man diagnosed with spinal gout, which led to a destructive deformity in the lumbar region. The condition was addressed through a two-stage circumferential reconstruction procedure, including oblique lumbar interbody fusion first, followed by endoscopic decompression utilization unilateral biportal endoscopy technology and transpedicular instrumentation fixation. After surgery, the patient got normal ambulation with successful fusion at the involved segment. This report highlights the surgical strategy for addressing spinal deformity associated with gout, while also advocating for appropriate treatment strategy tailored to this rare condition.

## Introduction

Spinal gout represents an uncommon presentation of gout, distinguished by the accumulation of monosodium urate crystals (tophi) within various spinal components, such as the vertebral bodies, intervertebral discs, and ligamentum flavum (1, 2). These crystalline deposits can trigger localized inflammation or mechanical compression, leading to a spectrum of neurological deficits including radiculopathy, myelopathy, or cauda equina syndrome (3, 4).

While pharmacological urate-lowering therapies remain the cornerstone of systemic management, surgical intervention becomes necessary when structural instability or neurological comprise occurs (5). However, current literatures lacks standardized protocols for complex cases of spinal gout. Specifically, there is a significant paucity of evidence regarding the application of minimally invasive surgery for circumferential reconstruction in the context of gout-induced vertebral destruction (6, 7). To address this gap in the literature, we report a rare case of gout-related lumbar destructive deformity successfully managed through a circumferential reconstruction.

## Case report

**Figure 1:**
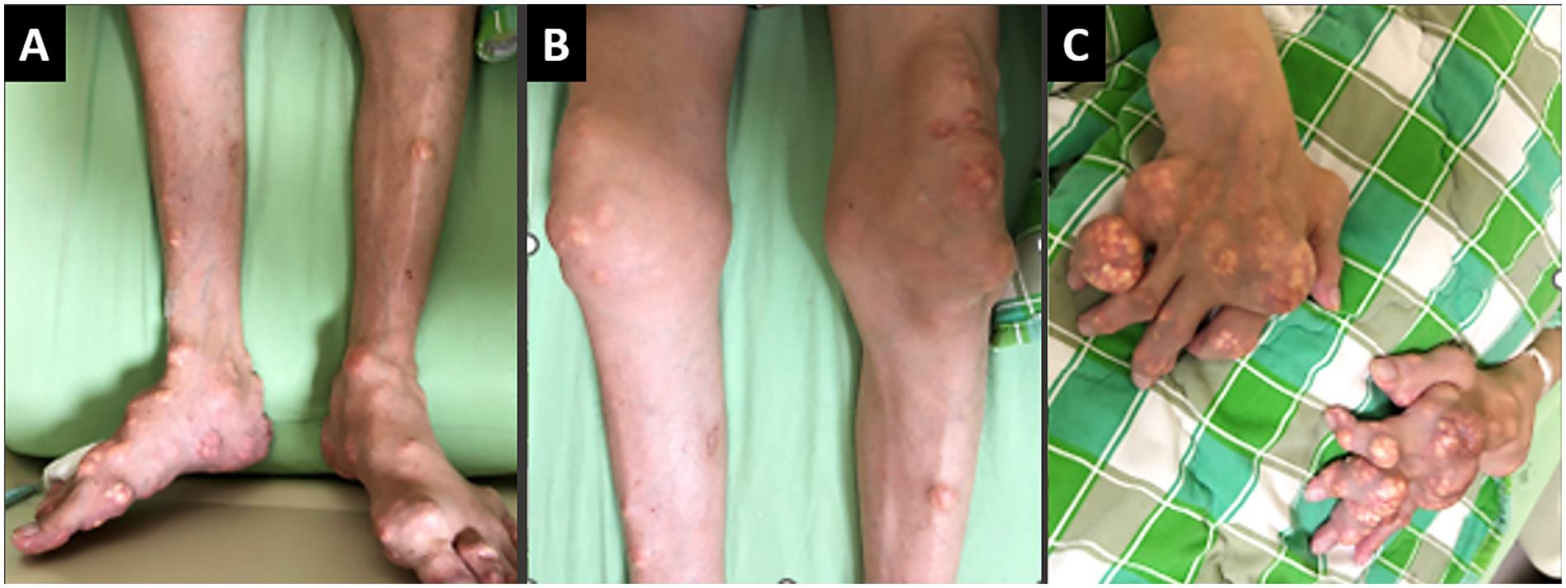
Severe tophi deposits in the patient's joints of feet **(A)**, knees **(B)**, and hands **(C)**

A 59-year-old male with a documented history of chronic gouty arthritis, who had not been using urate-lowering therapy due to drug hypersensitivity, presented with bilateral radicular discomfort in the lower back and manifestations of neurogenic claudication that had endured for a duration of two months. Additionally, he conveyed the presence of pain in both his hands and feet. A comprehensive physical examination disclosed multiple firm, nodular swellings, recognized as tophi, located on his bilateral wrists, fingers, knees, ankle, and toes (Figures 1A–C). Further assessment revealed tenderness along the midline and paracentral regions over the L3 and L4 vertebrae, diminished ankle reflexes bilaterally, and a decreased sensation to pinprick in the L3–4 dermatomes. Laboratory investigations corroborated the diagnosis of hyperuricemia, with an initial uric acid concentration documented of 9.6 mg/dL and a normal creatinine level of 1.1 mg/dL.

**Figure 2:**
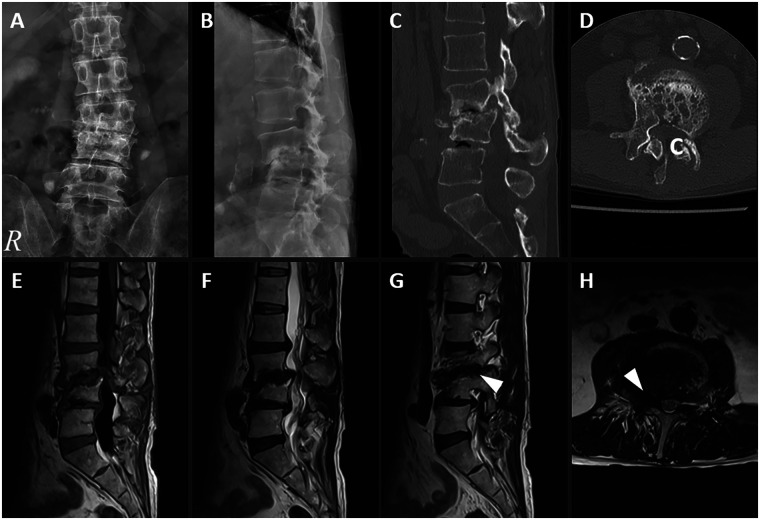
Preoperative imaging studies of the lumbar spine. Plain radiography **(A,B)** and computed tomography imaging **(C,D)** of the lumbar spine showed destructive erosion of endplates, lytic lesions over vertebral bodies and spondylolisthesis at the L3−4 spinal segment. Magnetic resonance imaging showed anterior slippage of the L3 vertebrae over L4 **(E,F)**, and posterior herniation of disc with severe stenosis (arrowhead) of the right L3−4 intervertebral foramen **(G,H)**.

Plain radiography and computed tomography imaging of the lumbar spine demonstrated destructive erosive changes of the endplates, lytic lesions affecting the vertebral bodies, and spondylolisthesis at the L3–4 spinal segment (Figures 2A–D).

**Figure 3:**
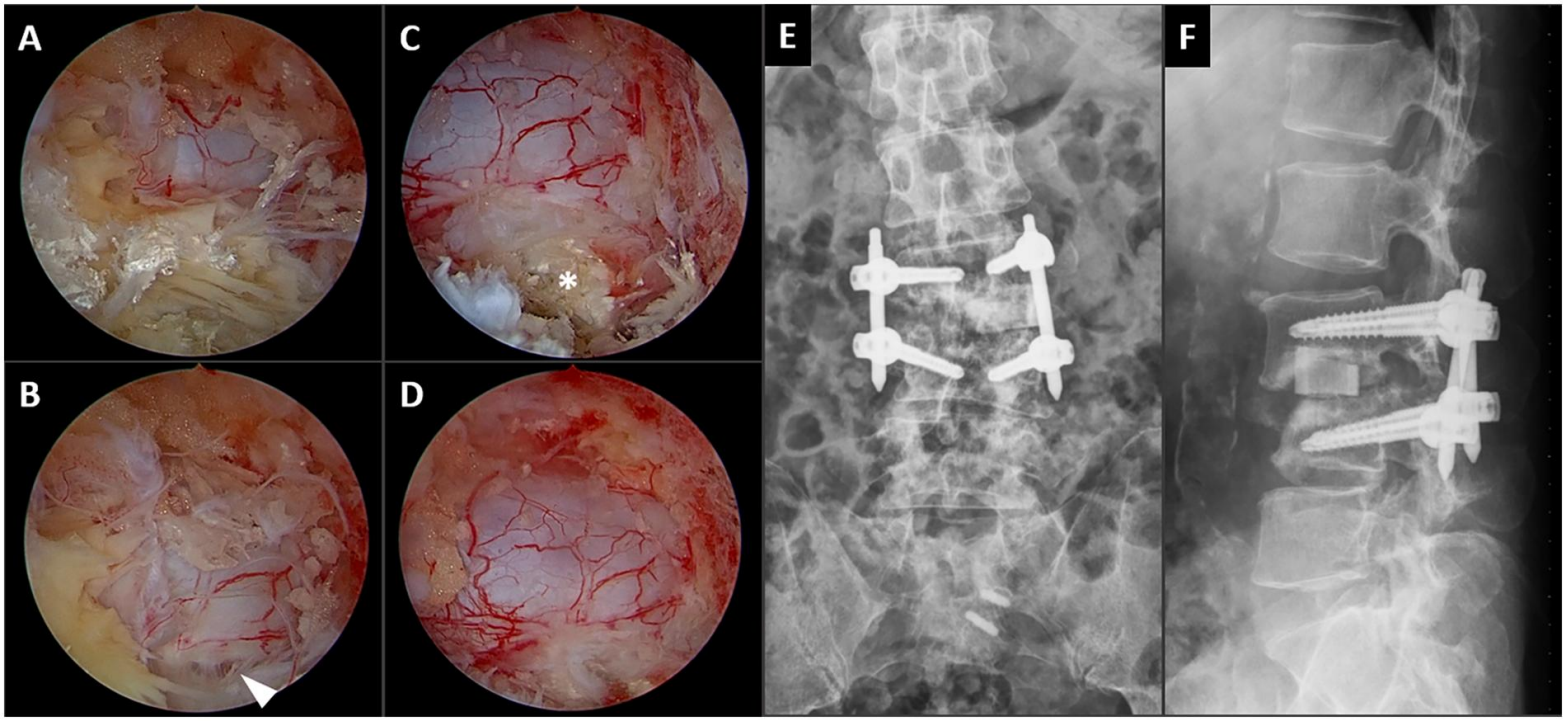
Intraoperative endoscopic images and postoperative plain radiography findings. Endoscopically, tophi material was observed mixed with the ligamentum flavum **(A)**, and the complex was tightly adherent (arrowhead) to the dura mater **(B)**. The herniated disc (asterisk) resulted in impingement of the right existing L3 nerve root **(C)**. After completing the decompression, the right existing L3 nerve root and right transverse L4 nerve root got decompressed **(D)**. The postoperative plain radiography showed that the kyphotic deformity of the L3−4 lumbar segment was corrected **(E,F)**, and the image three months later revealed that the L3−4 lumbar segment was well fused.

Magnetic resonance imaging (MRI) revealed a posterior herniation of the intervertebral disc, accompanied by severe stenosis of the right L3–4 intervertebral foramen (Figures 2E–H). A two-stage circumferential minimally reconstruction was employed (Figure 3).

Stage one involved a lateral retroperitoneal pre-psoas approach with allograft interbody fusion to correct coronal and sagittal alignment. Stage two achieved direct neural decompression via a unilateral biportal endoscopic interlaminar approach, completed with transpedicular instrumentation fixation. Postoperatively, the patient was able to engage in normal ambulation with a notable improvement in neurological deficits. Three months post-surgery, a plain radiograph of the lumbar spine illustrated successful fusion at the L3–4 lumbar segment.

## Discussion

Spinal gout can impact multiple anatomical components of the spine, such as the vertebral bodies, intervertebral discs, facet joints, ligamentum flavum, and the epidural space. The accumulation of urate crystals in these locations may result in mechanical compression of neural structures, inflammation, or a combination of both, leading to a spectrum of clinical presentations (3). Patients may exhibit localized back pain, radiculopathy, myelopathy, or indications of spinal stenosis. In more severe instances, neurological impairments such as motor weakness, sensory deficits, or dysfunction of bowel and bladder control may arise, contingent upon the particular spinal segment and degree of involvement (4). These manifestations frequently resemble other spinal disorders, rendering early diagnosis both challenging and imperative to avert irreversible neurological injury. The overarching objectives of surgical intervention encompass the decompression of neural structures, the restoration of spinal stability, and the alleviation of pain attributable to tophaceous accumulations (5, 6). The selection of surgical methods and strategies is contingent upon the specific location and severity of the disease, as well as the patient's overall health status.

Decompression procedures are frequently executed to alleviate pressure exerted on the spinal cord or nerve roots. If tophaceous deposits are located within the anterior spinal structures, an anterior surgical approach might be necessary for their effective removal. In scenarios where erosion or destruction of bone due to gout compromises spinal stability, spinal fusion is usually recommended. This procedure entails the stabilization of the affected spinal segment through bone grafting, and, when deemed necessary, the incorporation of instrumentation fixation. This approach aims to ensure enduring stability and mitigate the risk of further deformity or mechanical complications.

Accompany with flourish development of minimally invasive spinal technique, it can manage most degenerative and infectious spinal diseases with satisfactory outcome (8, 9). In the recent time, its clinical application for spinal gout is getting more emphasis. Our case highlights the circumferential minimally invasive surgery for reconstruct the spinal deformity secondary to spinal gout. With the swift advancement of minimally invasive spinal methodologies, particularly endoscopic spinal surgery, this approach demonstrates a substantial efficacy in managing a majority of degenerative spinal pathologies while yielding commendable outcomes. Recently, there has been a burgeoning interest in its clinical application for the treatment of spinal gout (7). This report delineates a case involving the reconstruction of a secondary gouty deformity of the spine through the utilization of circumferential minimally invasive surgical techniques.

Beyond addressing structural and neurological concerns, meticulous intraoperative dissection and neural manipulation are essential to curtail inflammation and hemorrhage, as gouty tophi are frequently associated with marked inflammation of the surrounding tissues. Research demonstrates a significant correlation between the magnitude of surgical procedures and peripheral blood inflammatory markers. Tracking these accessible biomarkers allows for the quantification of physical stress, providing clinicians with a reliable tool to monitor surgical invasiveness (10). Postoperative management encompasses vigilant monitoring for infection, the assurance of spinal stability, and the initiation of urate-lowering therapy to avert recurrence. Surgical treatment for spinal gout has the potential to markedly enhance patient outcomes; however, it necessitates meticulous planning and collaboration between surgical and medical teams to maximize both immediate and long-term results.

## Conclusion

The combination of oblique lumbar interbody fusion and unilateral biportal endoscopic surgery constitutes a feasible, minimally strategy for reconstructing gout-related lumbar destructive deformities. This hybrid technique not only restores spinal alignment but also allows direct neural decompression while minimizing soft tissue damage.

## Data Availability

The raw data supporting the conclusions of this article will be made available by the authors, without undue reservation.
